# Zebrafish *stm* is involved in the development of otoliths and of the fertilization envelope

**DOI:** 10.1530/RAF-20-0040

**Published:** 2021-02-16

**Authors:** Theeranukul Pachoensuk, Taketo Fukuyo, Md. Rezanujjaman, Klangnurak Wanlada, Chihiro Yamamoto, Akiteru Maeno, Md. Mostafizur Rahaman, Md. Hasan Ali, Toshinobu Tokumoto

**Affiliations:** 1 Integrated Bioscience Section, Graduate School of Science and Technology, National University Corporation, Shizuoka University, Suruga-ku, Shizuoka, Japan; 2 Biological Science Course, Graduate School of Science, Shizuoka University, Suruga-ku, Shizuoka, Japan; 3 Department of Animal Production and Fisheries, Faculty of Agricultural Technology, King Mongkut’s Institute of Technology Ladkrabang, Bangkok, Thailand; 4 Division of Technical Service, Shizuoka University, Suruga-ku, Shizuoka, Japan; 5 Mammalian Genetics Laboratory, National Institute of Genetics, Mishima, Shizuoka, Japan; 6 Facility and Equipment Technical Unit, National Institute of Genetics, Mishima, Shizuoka, Japan

**Keywords:** starmaker, zebrafish, fertilization, fertilization envelope, otoliths

## Abstract

Using an *in vivo* assay, we selected 11 genes that were highly upregulated during the induction of ovulation in zebrafish using microarray analysis and RNA sequencing. The starmaker gene (*stm*) was one of these genes. Although *stm* has been previously reported to be involved in otolith formation during the early development of zebrafish, we detected its expression in eggs and showed that *stm* was related to fertilization by establishing an *stm* gene knockout strain using the CRISPR/Cas9 system. Further phenotypic analysis of *stm* knockout fish was conducted in this study. With a higher nonfertilization rate, the stm mutant strain showed an extremely low survival rate. Otoliths of *stm* homozygous mutant zebrafish showed abnormal morphology in embryos and adult fish. However, fish did not show any abnormalities in swimming behaviour in either embryos or adults. Stm proteins were detected on the chorion of ovulated eggs before spawning. Fibre-supported knob-like structures on the fertilization envelope (FE) also showed abnormal structures in *stm* mutants. The Stm protein is necessary for otolith formation, and a lack of Stm causes abnormal otolith formation. The partial defect of otolith formation does not cause defects in swimming behaviour. The Stm protein is expressed in the chorion and is responsible for the formation of fibre-supported knob-like structures on the FE. It was suggested that a lack of Stm caused a lower fertilization rate due to inadequate formation of the FE.

**Lay summary:**

In zebrafish, the protein Starmaker (Stm) was identified as having a role in ovulation. Stm is also known to be required for the formation of ear stones (otoliths) which are needed to keep the body in balance. Zebrafish lacking Stm were produced by genome editing. As expected, Stm-deficient fish formed abnormal otoliths. To investigate the role of Stm in ovulation, fertilization and early development, we tried mating of Stm mutants and observed their juveniles. Although no problem found in ovulation, we found low fertilization rate and abnormal structure of knob-like structure (small pit) on the egg membrane. Survival rate of embryos with abnormal egg membrane was extremely low. It was demonstrated that Stm protein is necessary to form the functional egg membrane to protect embryos from the outside environment.

## Introduction

By using an *in vivo* induction method for oocyte maturation and ovulation in zebrafish by adding compounds to water (Tokumoto *et al.* 2011), we identified 11 genes that were highly upregulated during the induction of ovulation (Klangnurak *et al.* 2018). Then, we determined the functions of these genes by establishing gene knockout zebrafish strains of these genes. We have already established genome-modified fish for the starmaker (*stm*) gene. The *Starmaker* (*stm*) gene has been reported to be responsible for the formation of otoliths in zebrafish (Sollner *et al.* 2003).

Otoliths are important structural organs for equilibrium maintenance. Teleost fish, including zebrafish, possess a set of three types (conglomerate: Lapillus, flat stone: Sagitta, stellate: Asteriscus) of otoliths on each side of the body. Thus, there are six otoliths in each fish body (Platt 1993). Otoliths contain calcium carbonate and some matrix proteins, among which Stm is known to play an important role in changing the crystal structure of calcium carbonate from calcite to aragonite (Sollner *et al.* 2003). It is known that there are three types of crystal structures of calcium carbonate, calcite, aragonite, and vaterite, and calcite is the most stable crystal state at normal temperature and pressure. In mammals, calcite is the main component of otoliths, but in fish, aragonite is the main component. The stm protein is an acidic protein containing a large amount of aspartic acid, and some of the serine and threonine residues contained therein are phosphorylated. It is believed that this causes strong binding to calcium ions, which in turn causes a change in crystal structure from calcite to aragonite (Kalka *et al.* 2019).

Fish eggs develop a fertilization envelope (FE) that surrounds embryos and protects them from direct exposure to the outside water environment (Laale 1980, Grierson & Neville 1981, Harvey *et al.* 1983, Cameron & Hunter 1984). A perivitelline space is formed after fertilization by the cortical reaction on the vitelline membrane (Ohta & Nashirozawa 1996). The formation of this perivitelline space is related to blastodisc formation. The liquids between the vitelline membrane and the FE protect the egg against physical impacts with the external environment and are responsible for a gas exchange through diffusion (Donovan & Hart 1986). There have been no reports on the relationship between Stm and FE formation. In this study, we demonstrated that Stm is responsible for the proper formation of FE.

Although the fertilization rate decreased, ovulation could be induced in our *stm* homozygous mutant fish, which demonstrated that *stm* was not an ovulation-inducing gene (Klangnurak *et al.* 2018). In this study, further analysis of the *stm* mutant strain was conducted. Abnormal otolith shapes similar to the previous report of the knockdown experiment with antisense morpholino oligo was observed (Sollner *et al.* 2003). However, abnormal otolith morphology, which is important in fish equilibrium, had no effect on swimming behaviour. We found abnormal formation of fibre-supported knob-like structures on the FE that might be responsible for the hardening of the FE.

## Materials and methods

### Materials

Zebrafish (*roy*) were cultivated following the standard protocol (Westerfield 1995) and were kept in a flow system maintained at 28.5°C with a 14:10 light/dark cycle. Zebrafish were fed *Paramecium* spp. in the larval period for approximately 1 month, after which they were fed live brine shrimp in the morning and instant food (Tetra Guppy, Tetra GmbH, Melle, Germany) in the evening. In this study, the use of zebrafish and the experimental protocol for use were approved (approval no. 2019F-5 and 2020F-4) by the Institutional Ethics Committee of Shizuoka University, Japan.

### Mutant line generation and phenotype observation

The *stm* genome-edited fish were established using CRISPR/Cas9 (Klangnurak *et al.* 2018). The F0 zebrafish were investigated for mutations using heteroduplex mobility assay (HMA) (Foster *et al.* 2019). Heterozygous mutant zebrafish of *stm* (*stm*^+/−^) were established by pairing F0 mutant zebrafish and WT zebrafish. The same strain of *stm*^+/−^ was inbred to produce a homozygous mutant (*stm*^−/−^) in the F2 generation. The fertility of *stm*^−/−^ was investigated by pairing the zebrafish with each other or with WT fish. In the evening, *stm*^−/−^ males and females were placed in the breeding tank, which has a small cage inside the large tank. The bottom of the small tank has a net through which the fertilized eggs can pass through to the floor to prevent the adult fish from eating them. The next morning, the embryos were collected in the petri dish from the large tank. The embryos were counted, and the morphological characteristics of unfertilized, abnormal and surviving embryos were checked under a stereomicroscope. The fry (5 dpf) were released to the small tank and fed *Paramecium* spp. for at least 2 weeks. Then, the fish were fed live brine shrimp in the morning and instant food (Tetra Guppy, Tetra GmbH, Melle, Germany) in the evening. Strains of the *stm*^−/−^ mutant were propagated by pairing zebrafish with the same genotypes with each other. However, when no fertile females were obtained, heterozygous or WT females were used for pairing to continue the strain.

### DNA sequencing

Genomic DNA was amplified, which covered the target site of the *stm* gene for crRNA design, using the specific primers below. A 25 μL reaction mixture contained 0.5 μL (0.5 U) of KOD-Plus DNA polymerase, 2.5 μL of 10× Buffer for KOD-Plus, 2.5 μL of 2 mM dNTP mix, 1 μL of 25 mM MgSO_4_ (TOYOBO CO., LTD., Osaka, Japan), 0.75 μL each of 10 μM forward (5’-GACGTACAAGTGGAAGTAACTCTGG-3’) and reverse primer (5’-TGCTGTAACTCCTGTAATCTTTTCC-3’), and 4 μL of genomic DNA template. PCR was performed under the following conditions: 95°C for 2 min, three steps for 35 cycles of 95°C for 30 s, 58°C for 30 s, and 72°C for 1 min, and finally at 72°C for 10 min. PCR products were purified by alkaline phosphatase (AP) and exonuclease I (GE Healthcare Life Science). Five microlitres of DDW, 0.05 μL each of AP and exonuclease, and 10 μL of PCR product were added. The mixtures were incubated at 37°C and 80°C for 15 min. DNA sequencing was outsourced to Fasmac Co., Ltd. The DNA sequence analysis was performed using Codon Code Aligner (http://www.codoncode.com/aligner/) and GENETX-MAC (Ver.14.0.3).

### Immunohistochemistry

Before spawning, female zebrafish containing ovulated eggs on the posterior side of the abdomen were sacrificed by cervical spine destruction and fixed in 4% paraformaldehyde (PFA) at 4°C overnight and soaked with 30% sucrose for 3 h or until the samples sunk. The fixed fish body was transferred to an embedding chamber and embedded with Tissue-tek O.C.T. compound. The embedding chamber was dipped into liquid N_2_. Samples were cut into 10 μm thick sections on a cryostat microtome (CryoStar NX70, Thermo Fisher Scientific) at −20°C (Westerfield, 1995). The cut samples were transferred to slides and a PAP pen (Daido Sangyo Co. Ltd., Tokyo, Japan), which disperses a hydrophobic material, was used to circumscribe the section. The slides were gently washed three times with TPBS (0.1% Tween 20 in PBS solution; 0.8% NaCl, 0.02% KCl, 0.02 M PO_4_, pH 7.3) for 2 min. The solution was removed and replaced with blocking solution (5% nonfat milk in PBS buffer; 0.8% NaCl, 0.02% KCl, 0.02 M PO_4_, pH 7.3) for 30 min under a dark cover with distilled water-moistened paper. The slides were washed with TPBS for 5 min three times and incubated with anti-zebrafish starmaker MAB (Abmart Inc. Shanghai, China) diluted 100-fold in PBS at 4°C overnight in the dark. The samples were washed with TPBS for 10 min three times and the antibody was replaced with Alexa Fluor 555-conjugated anti-mouse immunoglobulin (Cell Signaling Technology) for 1 h at room temperature in the dark. The experimental slides were washed with TPBS 10 min three times, treated with prolonged reagent, and observed by confocal laser microscopy (LSM700, Carl Zeiss).

### Stereoscopic observation

A stereomicroscope (Olympus SZX12) and a microscope camera (Olympus DP70) were used for observation and photography. The otoliths of WT and stm mutant fry were observed and photographed under a microscope. Otoliths (pebble stones: Lapillus, flat stones: Sagitta, star stones: Asteriscus) were excised from the WT and stm mutant adult fish and photographed.

### Scanning electron microscope observation

Otoliths (pebble stones: Lapillus, flat stones: Sagitta, stellates: Asteriscus) were extracted from adult WT and stm^−/−^ mutants. The FEs were removed from eggs in water and washed with ultrapure water. Eggs with FEs were washed with ultrapure water. Samples were washed with ultrapure water and then critical point dried using a freeze dryer (Aqua FD-6500; SUN Technologies, Kyoto, Japan). Dried samples were coated with 5 nm platinum using an autofine coater (JEC-3000FC; JEOL, Tokyo, Japan). Subsequently, the FEs were observed with s.e.m. (JSM-6510LV; JEOL).

### Micro-CT imaging

WT and stm mutant adult zebrafish were fixed with 70% ethanol and stored in 70% ethanol. The heads of zebrafish were scanned using an X-ray micro-CT device (ScanX- mate-E090S105, Comscantechno Co., Ltd., Japan) at a tube voltage peak of 60 kVp and a tube current of 100 μA. The sample was rotated 360° in steps of 0.24°, generating 1500 projection images of 992 × 992 pixels. The micro-CT data were reconstructed at an isotropic resolution of 13.3 × 13.3 × 13.3 μm. Three-dimensional tomographic images were obtained using OsiriX MD software (version 9.0, Pixmeo, SARL, Switzerland) and Imaris software (version 9.1, Carl Zeiss Microscopy Co., Ltd.). Supplementary video (Supplementary video 1, see section on supplementary materials given at the end of this article) was edited using Adobe Premiere Pro CC (Adobe Systems Co., Ltd., Japan).

### Statistical analysis

Summary data are presented as the mean ± s.d. Student’s *t* test was used to determine the statistical significance of the difference in data. Data were considered significant at *P < 0.05 or **P < 0.001.

## Results

**Figure 1:**
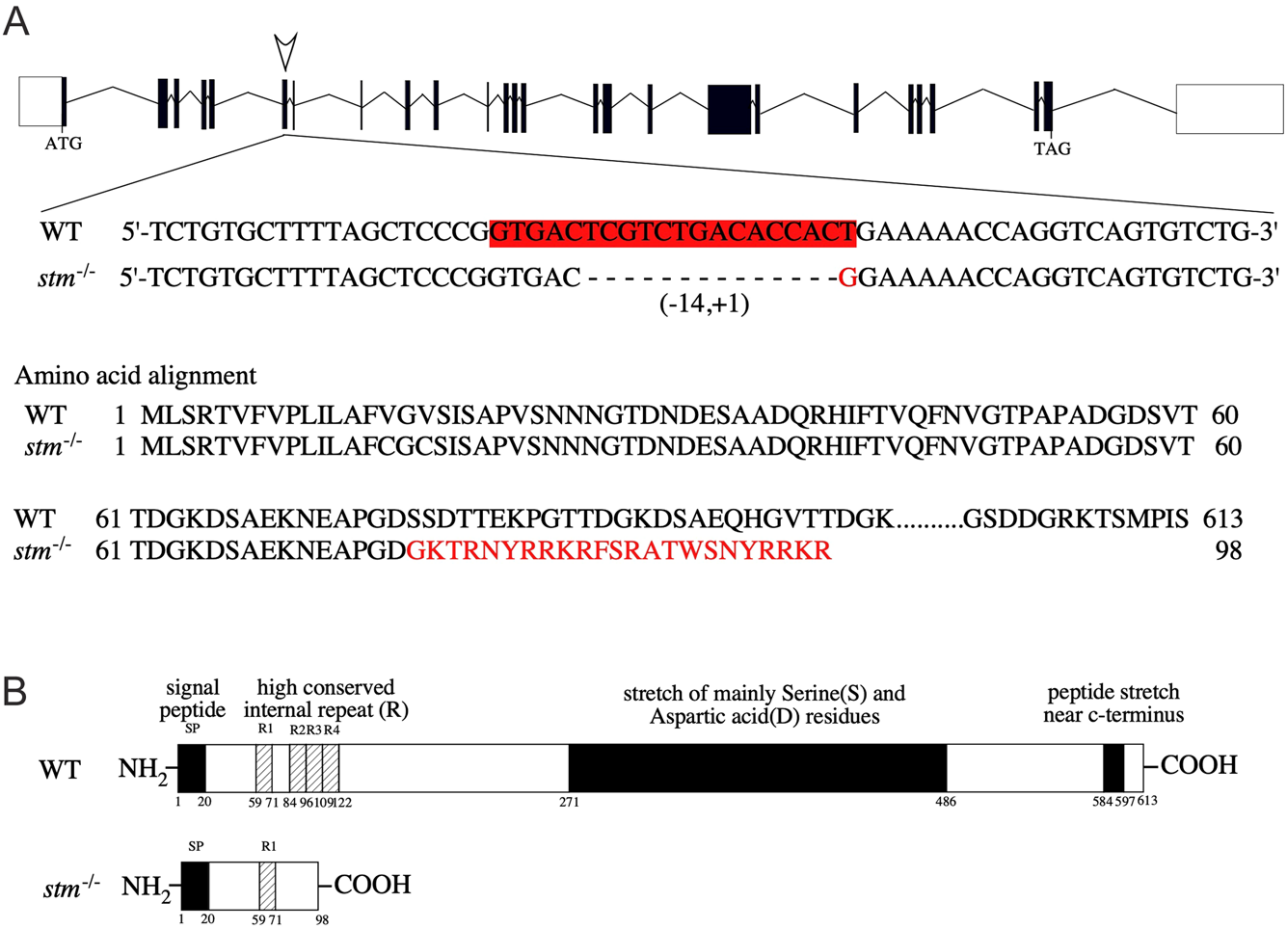
DNA sequence and predicted protein structure of an established stm gene-edited strain. (A) Genome structure and DNA sequence of the target site for the genome editing of *stm*. DNA sequences around the target site (in red) for CRISPR/Cas9 digestion in WT and *stm*^−/−^ mutants are indicated. A 14-nucleotide deletion and 1-nucleotide insertion were induced in the target site in the selected mutant. (B) The predicted protein structures of the WT and the *stm*^−/−^ mutant are indicated. It is expected that a peptide of 98 amino acids in length with an N-terminal 76-amino acid sequence is the same as WT Stm and is produced in the *stm*^−/−^ mutant. Thus only first high conserved internal repeat (R1) is present in Stm protein produced in the *stm*^−/−^ mutant.

**Figure 2:**
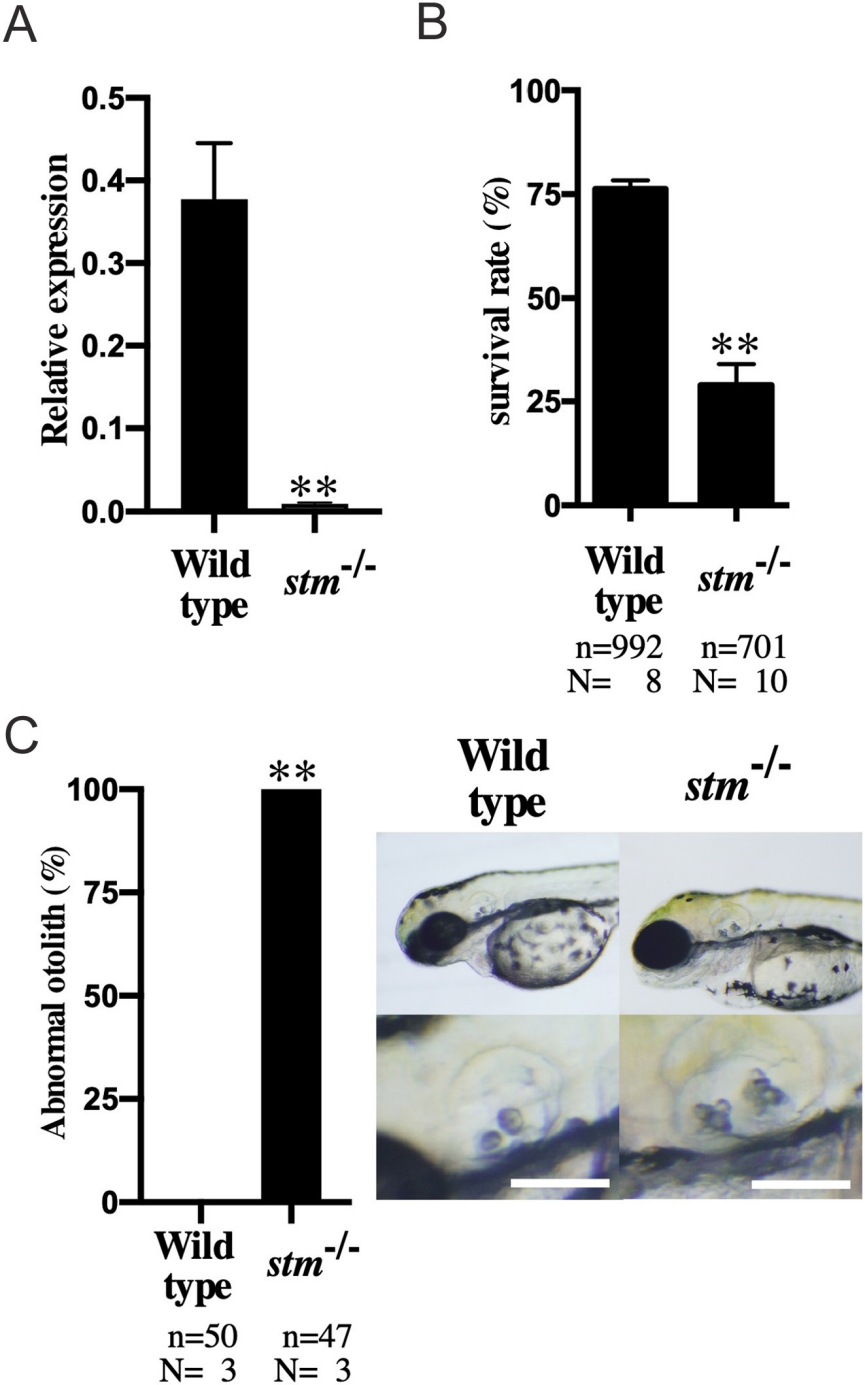
Expression of stm, survival rate and abnormalities of otoliths in *stm*−/− mutant. (A) Relative expression of *stm* in embryos of WT and *stm*^−/−^ mutant were compared. mRNA abundance was measured in triplicate for each sample from three different paring, and all data were normalized by the number of elongation factor 1α (EF1α) transcripts in each sample. (B) Survival rates of embryos in the WT and *stm*^−/−^ mutant (F6 generation) at 5 dpf. *n* = total number of embryos; *N* = number of biological replicates (embryos from different pair of fishes). (C) Percentages of abnormal otolith containing embryos in the WT and *stm*^−/−^ mutant. *n* = total number of embryos; *N* = number of biological replicates (embryos from different pair of fishes). Representative otoliths of 5 dpf embryos in WT and *stm*^−/−^ mutant are indicated. Scale bars are 100 µm. Asterisks represent significant difference between the samples (**P ≤ 0.001).

A mutant strain of the *stm* gene with deletion of 14 bps and insertion of 1 bp within exon 7 was found (Fig. 1A). While the WT zebrafish Stm protein is 613 amino acids in length, it was presumed that the *stm^−/−^* mutant expressed the peptide of 98 amino acids in length with the same sequence of the first 76 amino acids as the WT (Fig. 1B). However, significantly lower expression of *stm* mRNA was detected in *stm^−/−^* mutants by qPCR analysis (Fig. 2A). Thus, it was suggested that even truncated form of Stm protein was not expressed in *stm^−/−^* mutants.

**Figure 3:**
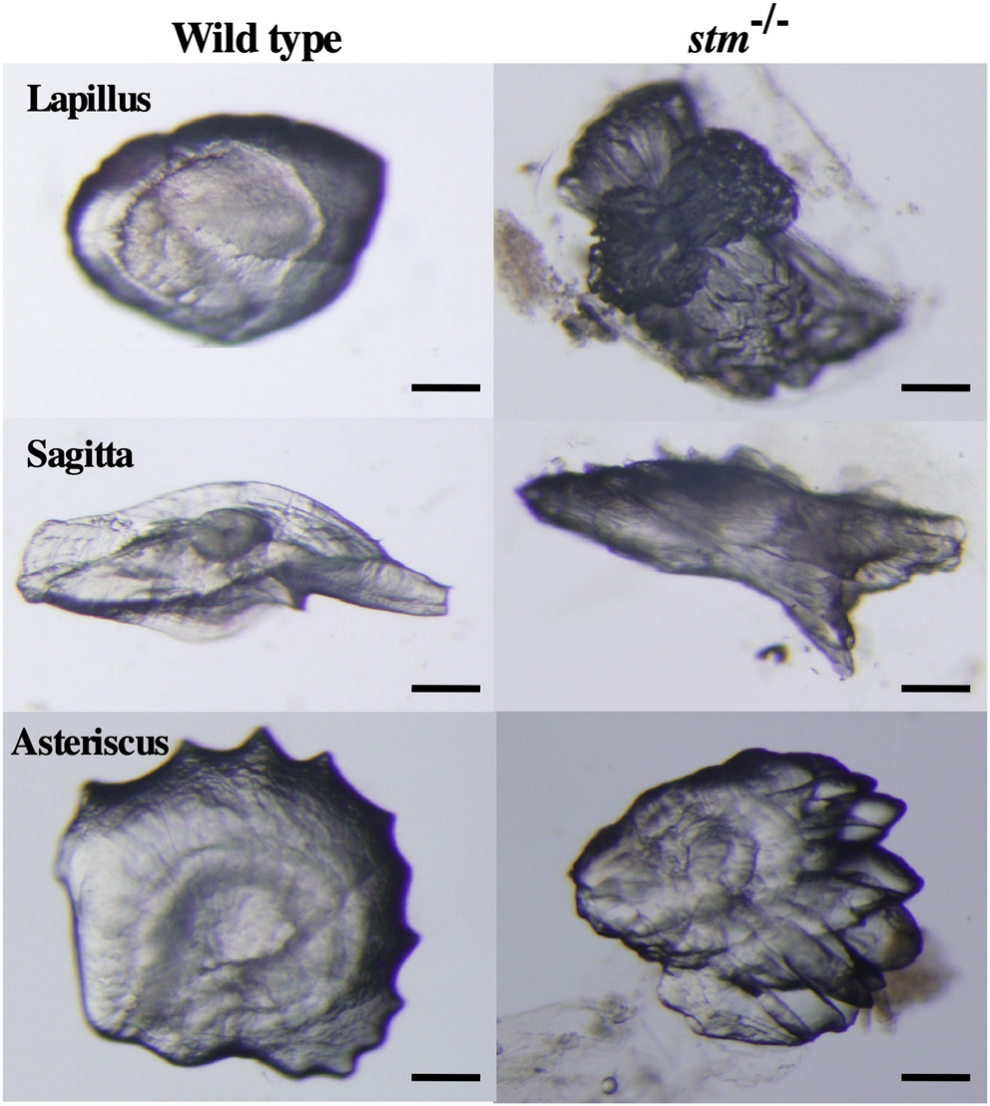
Stereomicroscopic observation of otoliths. Photographs of three excised otoliths (Lapillus, Sagitta and Asteriscus) from adult WT zebrafish and the *stm*^−/−^ mutant are indicated. Scale bars are 100 µm.

**Figure 4:**
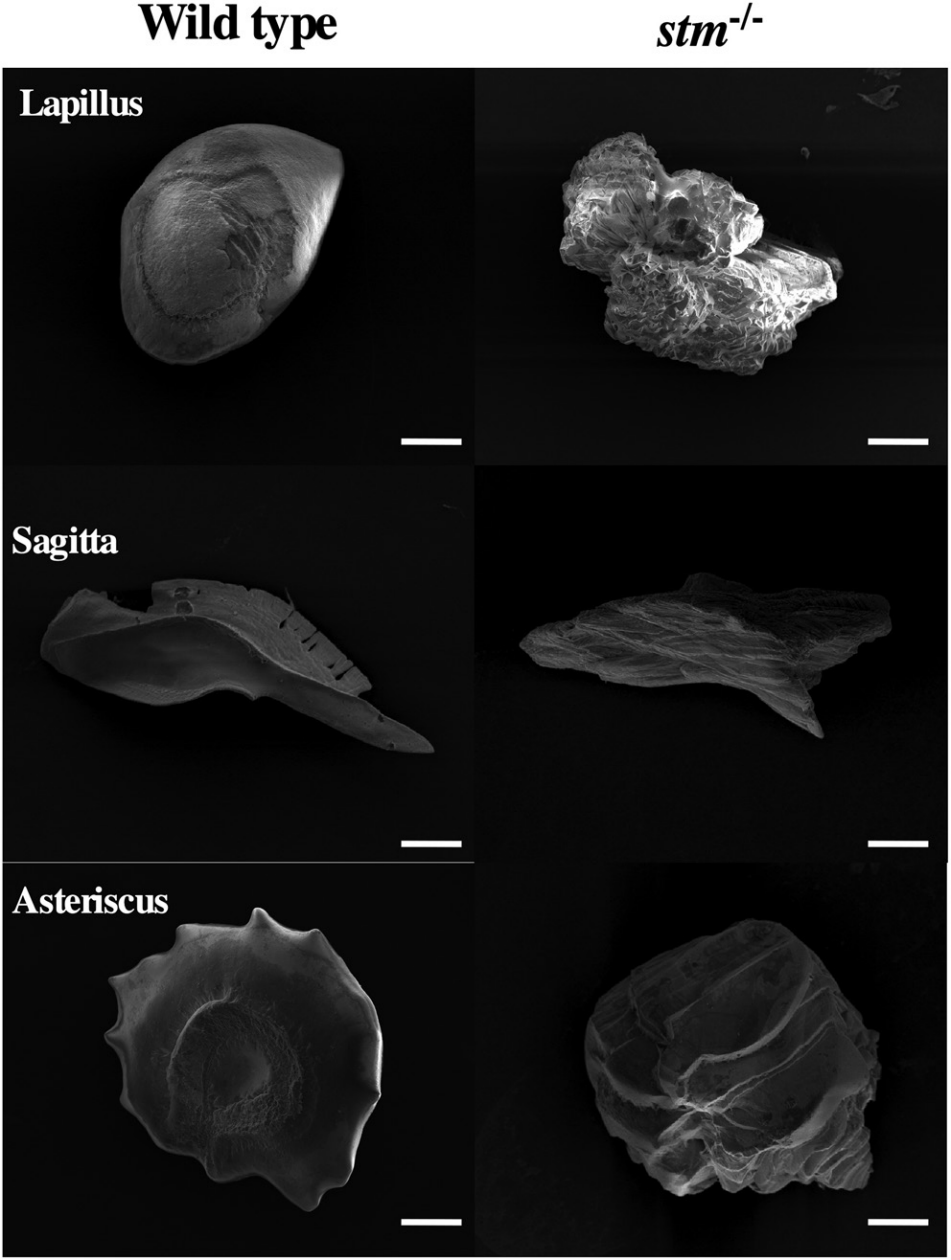
Observation of otoliths by SEM. Photographs from scanning electron microscopy observations of three excised otoliths (Lapillus, Sagitta and Asteriscus) from adult WT zebrafish and the *stm*^−/−^ mutant are indicated. Scale bars are 100 µm.

Because we selected *stm* gene as an ovulation-relating gene that showed significant increase of mRNA expression during ovulation. Thus, it is highly possible that mutations in *stm* gene show maternal effects. Then we used embryos and fishes obtained from paring of *stm^−/−^* mutants in all the phenotypic analysis below. The survival rate of embryos was significantly low in embryos from the pairing of *stm^−/−^* mutants due to the high nonfertilization rate (Fig. 2B). Two otoliths were observed in both WT and mutant embryos (Fig. 2C). In the mutants, star-shaped otoliths, which gave rise to the gene name (*starmaker*), were observed in all the embryos examined. In the adult fishes, three types of otoliths, pebbles (Lapillus), flat stones (Sagitta), and stellates (Asteriscus), developed in the *stm^−/−^* mutant and the WT fish. However, the shape of these otoliths was abnormal in all the *stm^−/−^* mutants fishes examined (more than five of females and males) (Fig. 3). The shape of the Lapillus in the *stm^−/−^* mutant was significantly different from that in the wild type. Lapillus in the WT showed a smooth surface but the surface in the *stm^−/−^* mutant was rough. Although relatively similar shapes were observed in the case of Sagitta and Asteriscus, the surfaces of these stones were also uneven in the *stm^−/−^* mutant compared with the wild type. The differences were more clearly observed using scanning electron microscopy (s.e.m.) (Fig. 4). Pebbles appeared as crystal clusters in the *stm^−/−^* mutant. Sagitta and Asteriscus showed layered structures in the *stm^−/−^* mutant. In contrast, the surfaces of all three stones were smooth in the wild type.

**Figure 5:**
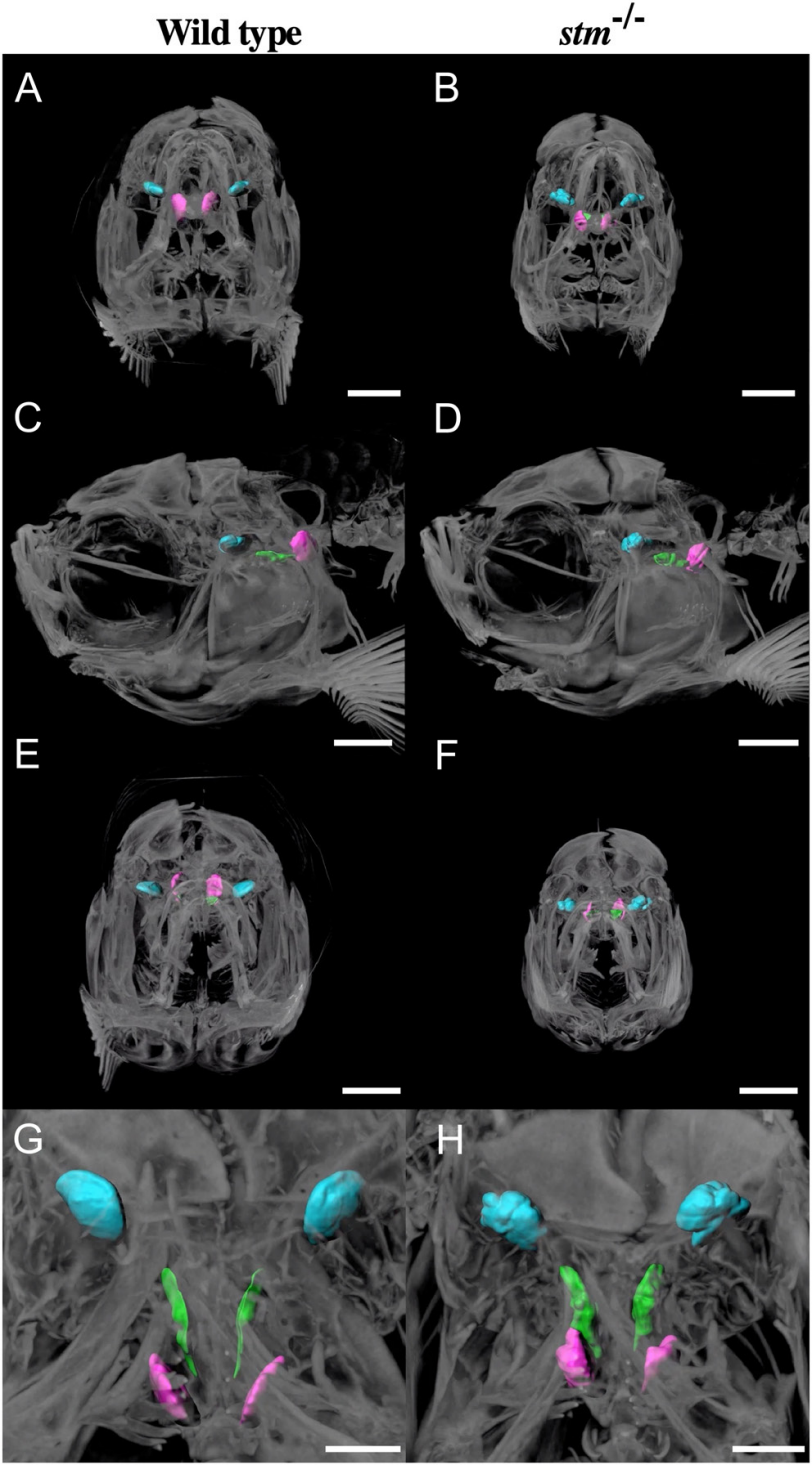
Observation of otoliths by micro CT. Micro CT scan images from anterior (A and B), left lateral (C and D), posterior (E and F) and dorsal (G and H) sides of adult WT zebrafish and the *stm* ^−/−^ mutant are indicated. Scale bars in A–F are 1 mm. Scale bars in G and H are 500 µm. Three otoliths are indicated in different colours: Lapillus; blue, Sagitta; green, Asteriscus; magenta. Scale bars are 400 µm.

Then, we tried to observe otoliths *in vivo* by micro-CT scanning (Fig. 5). Although the shapes of the three types of otoliths were different, the placement of otoliths was similar between the WT and the *stm^−/−^* mutant. The three-dimensional arrangement of otoliths in the WT and the *stm^−/−^* mutant can be compared in the Supplementary movie (sMovie 1). As mentioned earlier, the morphology of otoliths in the *stm^−/−^* mutant was abnormal, as expected. However, the *stm^−/−^* mutant zebrafish did not show any abnormality in behaviour (Supplementary videos 2 and 3). We tried to check the movement using tactile stimulation in embryos (Supplementary video 2) (Yang *et al.* 2011). Additionally, we checked female chasing behaviours during mating in adulthood (Supplementary video 3). We could not detect any difference in movement between WT and the *stm^−/−^* mutants. In tactile stimulation in embryos, all the embryos examined (total 70 embryos) from three different pairs of *stm^−/−^* mutants showed no abnormal behaviour. In adult, 7 of *stm^−/−^* male showed normal female chasing behaviours.

**Figure 6:**
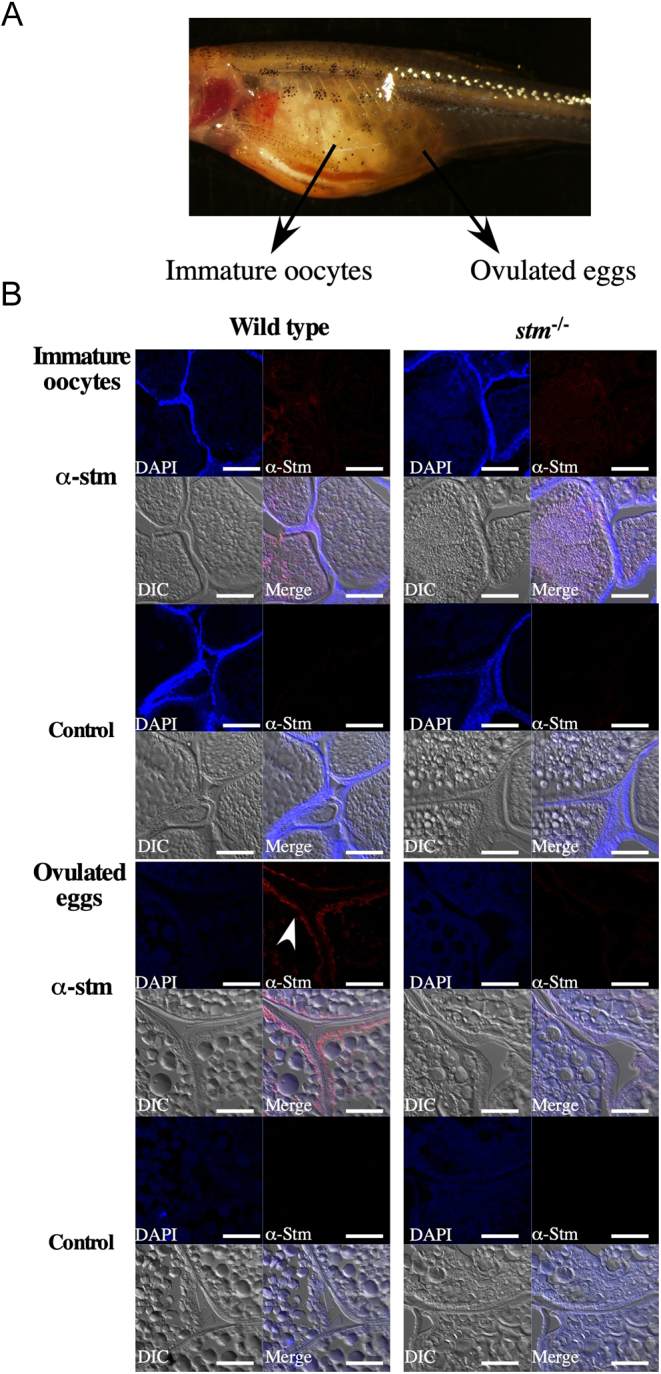
Immunohistochemical observation of Stm. (A) A photograph of *stm*^−/−^ mutant females possessing ovulated eggs. Frozen sections of immature oocytes and ovulated eggs were prepared from the fish. (B) Immunohistochemical staining results for Stm in WT and in *stm*^−/−^ mutant immature oocytes (upper panel) and ovulated eggs (lower panel). Frozen sections of the whole bodies of WT and *stm*^−/−^ mutant zebrafish females were stained with anti-Stm antibodies (α-stm) and DAPI. Differential contrast (DIC) images and merged images of anti-Stm, DAPI and DIC are also indicated (Merge). Control staining (Control) without anti-Stm antibodies of each sample is indicated below. The white arrow indicates signals in anti-Stm staining. The scale bars indicate 50 µm.

In addition to the morphology of otoliths, we had already found that eggs from *stm^−/−^* mutants showed a high nonfertilization rate (Klangnurak *et al.* 2018). By paring *stm^−/−^* mutants in later generations (F3 to F5), we obtained high percentages of unfertilized eggs. Thus, only a few juvenile survivors were obtained from the F3 generation and later generations. The same results were confirmed in the F6 generation (Fig. 2B). However, few survivors showed fertility, and we could continue the strain. To address the reason for unsuccessful fertilization, we conducted immunohistochemical staining of oocytes and eggs. In the sections of whole body sectioning of fish before spawning, which contained ovulated eggs and immature oocytes, strong Stm signals were detected on the chorion of ovulated eggs in WT (Fig. 6). In the sections of immature oocytes that were located in the anterior position in the ovary, the nuclei of follicular cells were stained with DAPI, but no Stm protein signal was observed. In the sections of ovulated eggs that were located in the posterior position in the ovary, signals of the binding of anti-Stm antibody were observed on the chorion. The absence of DAPI signals around the chorion confirmed the removal of follicular cells by ovulation. Contrary no signal of binding of anti-Stm antibody were observed on the chorion of ovulated eggs in *stm^−/−^* mutants. The results suggested that Stm was expressed during ovulation and accumulated on the surface of ovulated eggs. Furthermore, it was confirmed that the absence of Stm in *stm^−/−^* mutants.

**Figure 7:**
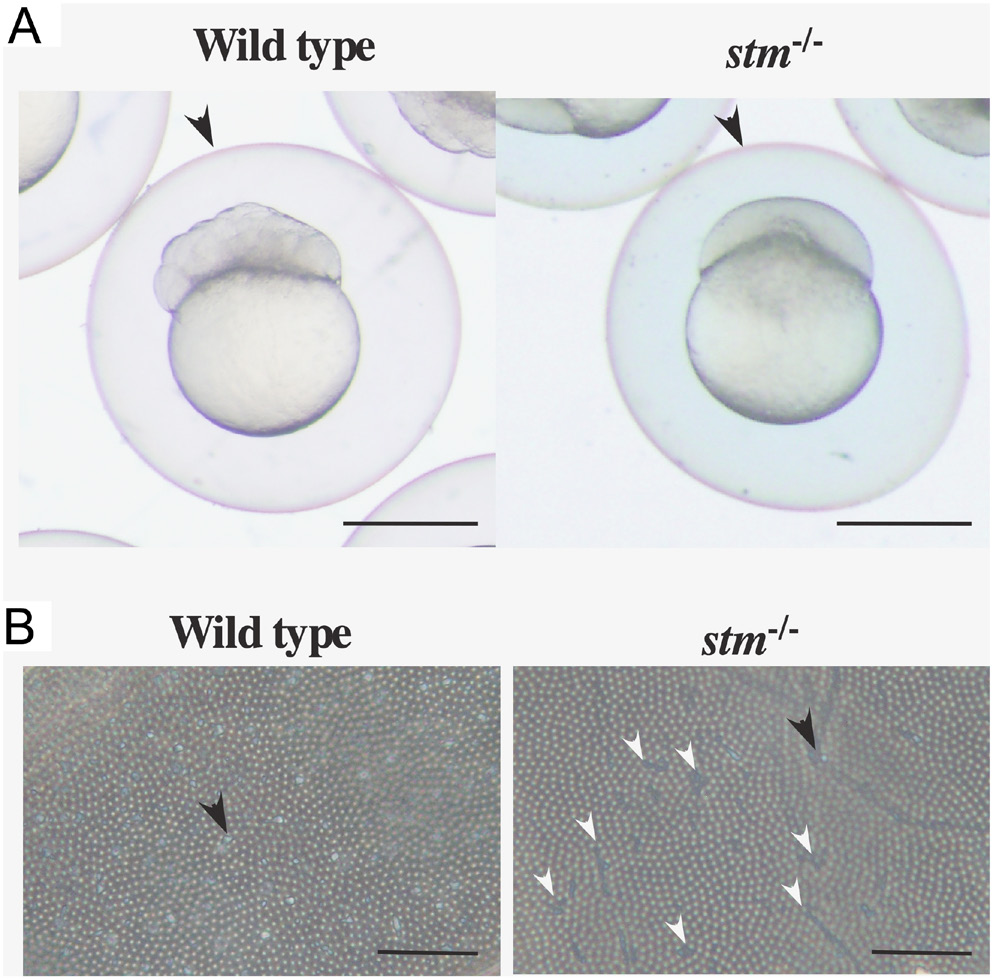
Observation of the FE. (A) Photographs of FE-covered embryos of WT and *stm*^−/−^ mutants. FEs are indicated by arrowheads. Scale bars are 500 µm. (B) Photographs from microscope observation of FE from WT and *stm*^−/−^ mutant embryos are indicated. A crystal-like structure is indicated by the black arrow. Cracks in the FE of* stm*^−/−^ mutants are indicated by white arrowheads. Scale bars are 20 µm.

**Figure 8:**
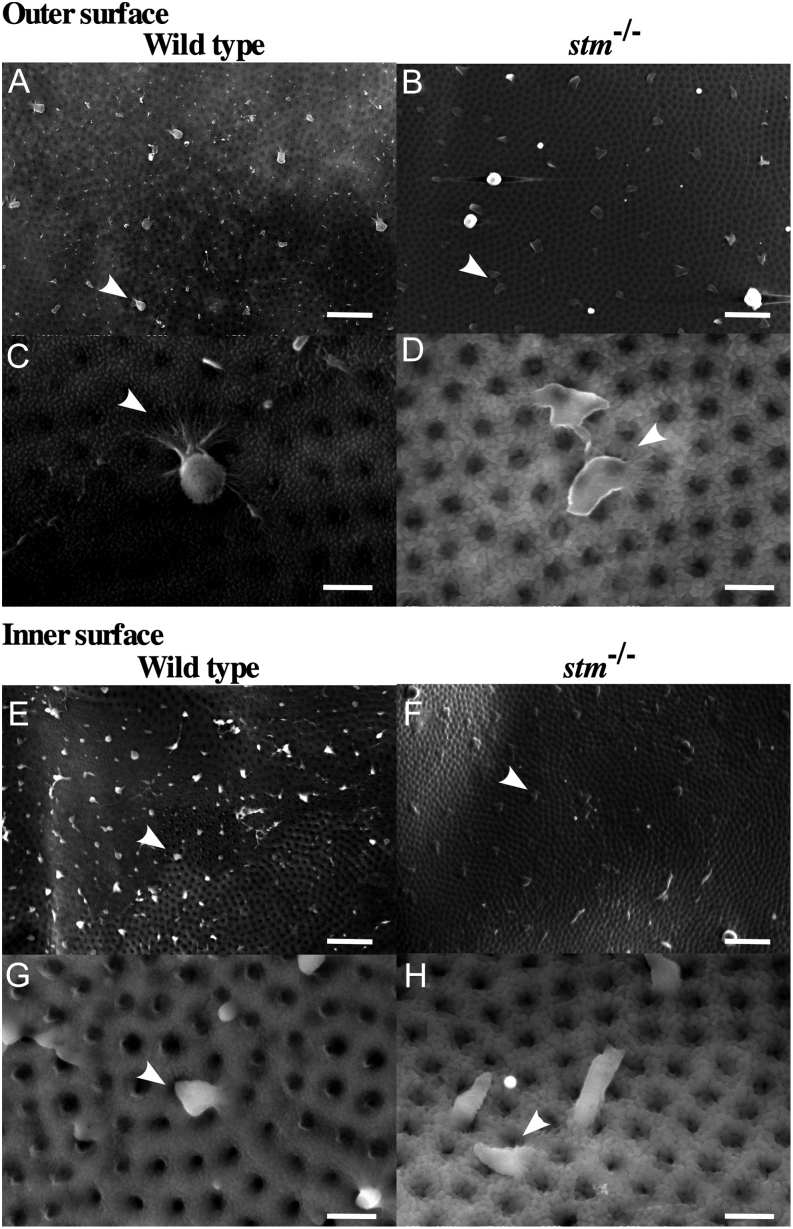
Observation of the FE by SEM. Photographs from scanning electron microscopy observations of the outer surface of the FE (A and B in 1500×; C and D in 8000×) and the inner surface of the FE (E and F in 1500×; G and H in 8000×) from adult WT zebrafish and the *stm*^−/−^ mutant are indicated. A fibre-supported knob-like structure is indicated by an arrow. Scale bars in A, B, E, F are 2 µm. Scale bars in C, D, G, H are 10 µm.

**Figure 9:**
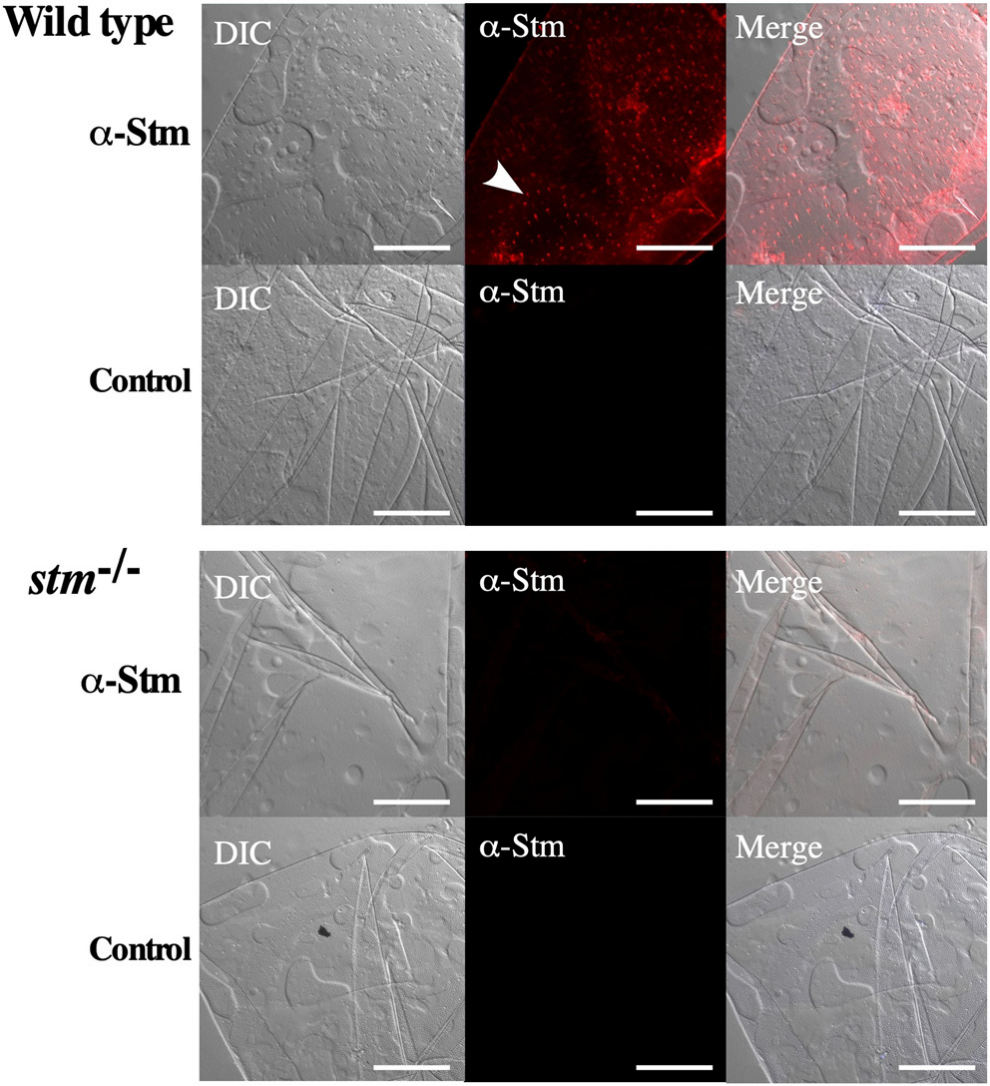
Immunohistochemical observation of Stm on FE. Immunohistochemical staining results for Stm of FE from WT and in *stm*^−/−^ mutant. FEs of WT and *stm*^−/−^ mutant zebrafish embryos were stained with anti-Stm antibodies (α-Stm). Differential contrast (DIC) images and merged images of anti-Stm and DIC are also indicated (Merge). Control staining (Control) without anti-Stm antibodies of each sample is indicated below. The white arrow indicates signals in anti-Stm staining. The scale bars indicate 50 µm.

From the expression of Stm on the chorion, it was expected that Stm plays a role in the formation of the FE. Thus, we observed the FE. On the surface of the FE, many crystal-like structures were found on the WT and only an extremely small number of structures were found on the FE of the *stm^−/−^* mutants by microscopic observation (Fig. 7). Additionally, there were many cracks in the FE of *stm^−/−^* mutants but not in the WT. For a more in-depth look at the crystal-like structures, we observed the FE by s.e.m. It was confirmed that the crystal-like structures on the surface of the FE were fibre-supported knob-like structures reported before (Joo & Kim 2013), and these structures were present on both sides of the FE (Fig. 8). In *stm^−/−^* mutants, knob-like structures appear to be crushed and spread. Presence or absence of Stm in knob-like structures in WT or *stm^−/−^* mutants were confirmed by the immunohistochemical staining of FE. Signals of the binding of anti-Stm antibody were observed in knob-like structures on the FE of WT (Fig. 9). On the contrary, no signal of binding of anti-Stm antibody were observed on the FE of *stm^−/−^* mutants. It was indicated that Stm is necessary for the formation of fibre-supported knob-like structures on FE.

## Discussion

In this study, phenotype analysis was conducted on a *stm* mutant strain with a different sequence from 77 aa to 98 aa. We observed structures that closely resembled the shape of otoliths in the fry of our mutant strain, which is similar to that of *stm* knockdown individuals injected with moderate amounts of MO (Sollner *et al.* 2003). Particular morphology of the star-like structure was found in Lapillus, the most anterior otolith among the three otoliths (Baxendale & Whitfield 2016). The location of otoliths in *stm^−/−^* mutants was close to that in WT. The *stm^−/−^* mutants did not show any deficiency in movements from embryo to adulthood. Thus, morphological changes in otoliths caused by the lack of Stm do not cause the problems in maintaining balance.

In this study, we found strong expression of Stm on the chorion. Additionally, we found that Stm is necessary for the formation of fibre-supported knob-like structures on the surfaces of the FE (Joo & Kim 2013). It was suggested that the formation of fibre-supported knob-like structures is necessary for a fully functional FE and that improper FE formation results in nonfertilization. We judged abnormal formation of single cells on the animal pole as unfertilized eggs. However, it is possible that the phenotype is also caused by the insufficient formation of the FE. These results suggested that Stm is necessary not only for otolith formation but also for the formation of the FE. The microscopic observation showed cracks in the FE. During the preparation of samples for s.e.m. observation, decompression treatment caused wrinkles in the FE of *stm* mutant eggs, whereas the FE of WT remained smooth. These results suggested that Stm may have a role in the hardening of the FE.

As the FE is a barrier for embryos from the outside environment, its permeability to substances has been analysed for a long time. As a result, it is generally accepted that the FEs shield the zebrafish embryo against solutes in the exposure solution, as it possesses pores of an average diameter of 1.5 μm that occur at distances ranging from 1.5 to 3 μm (Laale 1977, Rawson *et al.* 2000, Lee *et al.* 2007). Additionally, transparencies of toxic compounds were tested, and the most significant example was atrazine. Wiegand *et al.* have shown in uptake studies using ^14^C-labelled atrazine that atrazine penetrates the FE very quickly and reaches the zebrafish embryo within the first 10 s after the onset of exposure (Wiegand *et al.* 2000). Creton showed the passage of 3 kDa fluorescently labelled dextrans across the FE but a restriction of 10 kDa fluorescent dextrans (Creton 2004). More recently, Lee *et al.* established an *in vivo* imaging method to observe the entry of silver nanoparticles (Ag NPs) through the FE (Lee *et al.* 2007, 2012). They showed that single Ag NPs (30−72 nm diameters) passively diffused into the embryos through pores in FE via random Brownian motion and stayed inside the embryos throughout their entire development and caused dose- and size-dependent toxic effects. These results indicated that the FE is a sieve with an approximate pore size of 3000 Da. The most remarkable report concerning the function of the FE was the absorption of Cd^2+^. Burnison *et al.* demonstrated that 61% of the total absorbed Cd^2+^ was bound to the FE and that only a small proportion of 1% was found in the zebrafish embryo during a 4-h exposure by using ^109^Cd (Burnison *et al.* 2006). In our *stm* knockout zebrafish, the formation of fibre-supported knob-like structures was disrupted. From the known functions of Stm as a Ca^2+^-binding protein, it can be hypothesized that a lack of Stm caused a spreading form of knob-like structures. This caused a need to change the function of FE and resulted in changes in the components or conditions of the fluid. Further analysis of the composition of fluid and the strength and substance permeability of FE in *stm^−/−^* mutants are necessary for the future.

In *stm*^−/−^ zebrafish, mutants showed abnormal otolith structures. However, no obvious deficiencies in the movement were observed. We showed for the first time that Stm is involved in the formation of fibre-supported knob-like structures on FE. Although the functions of this structure remain to be solved, altering the strength and substance permeability of the FE might be the cause of unfertilized eggs.

## Supplementary Material

Video S1 Three-dimensional tomographic images of wild type and stm mutant adult zebrafish were obtained using the OsiriX MD software (version 9.0, Pixmeo, SARL, Switzerland) and Imaris software (version 9.1, Carl Zeiss Microscopy Co., Ltd., Japan). The video was edited using Adobe Premiere Pro CC (Adobe Systems Co., Ltd., Japan). Three otoliths are indicated in different colors, Lapillus; blue, Sagitta; green, Asteriscus; magenta. (mp4)

Video S2 Response of stm-/- mutant zebrafish embryos to touch with a glass tube. The stm-/- mutant zebrafish at 5 days post-fertilization with abnormal otoliths was touched with a glass tube, and its swimming behavior was observed. The bright-field videomicrograph was taken with an Olympus IX70-FLA inverted fluorescence microscope equipped with a Sony DCR-PC120 digital video camera. (mpg)

Video S3 Chasing behaviors during mating of stm-/- mutants. In the evening on the day before capture, stm-/- males and females were placed in the breeding tank, which has a small cage inside the large tank. The next morning, the video was taken with handy video (HC-W580M, Panasonic, Japan). (mov)

## Declaration of interest

The authors declare that there is no conflict of interest that could be perceived as prejudicing the impartiality of this study.

## Funding

This study was supported by a grant from JSPS KAKENHI (Grant Number 16K07419 and 20K06719) (to T T). This work was also supported by Grants-in-Aid for Scientific Research in Priority Areas from the Ministry of Educationhttp://dx.doi.org/10.13039/100010002, Culture, Sports, Science and Technology of Japan.

## Author contribution statement

T P drafted the manuscript. K W conducted genome editing. K W, T F, Md. R, T P, Md. M R and Md. H A established the strains and maintained them. T F and T P performed immunofluorescent observation. C Y, T P and T P conducted electron microscope observations. A M performed micro CT scan observations. T T participated in the study design, supervised the study and wrote the paper. All authors read and approved the final manuscript.
